# Radiographic findings have an association with weight bearing and locomotion in English bulldogs

**DOI:** 10.1186/s13028-020-00517-3

**Published:** 2020-05-12

**Authors:** Sari Helena Mölsä, Heli Katariina Hyytiäinen, Kaj Mikael Morelius, Maria Katariina Palmu, Tommi Sakari Pesonen, Anu Katriina Lappalainen

**Affiliations:** 1 Department of Equine and Small Animal Medicine, Faculty of Veterinary Medicine, University of Helsinki, P.O. Box 57, Viikintie 49, 00014 Helsinki, Finland; 2 4pharma Ltd, Tykistökatu 4D, 20520 Turku, Finland

**Keywords:** Brachycephaly, Conformation, Elbow dysplasia, Gait analysis, Hip dysplasia, Patellar luxation, Spinal malformation, Static weight bearing

## Abstract

**Background:**

English bulldogs are known to be prone to skeletal problems, but knowledge is lacking of the effect of these problems on locomotion and function. This study was undertaken to report the conformational, orthopaedic and radiographic findings in a cohort of English bulldogs in Finland and to evaluate how these findings affect weight bearing and locomotion of the dogs. Twenty-eight English bulldogs were prospectively recruited to this cross-sectional study. An orthopaedic examination, measurements of conformation, static and dynamic weight bearing, and radiographic examinations of elbow, hip, stifle joints and spine were done.

**Results:**

The English bulldogs carried a mean of 67.3% and 62.1% of their body weight in front limbs while standing and trotting, respectively. Front and hind limb lameness was seen in 20.8% (5/24) and 12.5% (3/24) of dogs, respectively. At orthopaedic examination, abnormal palpation findings (i.e. pain response, crepitation, swelling or subjectively decreased range of motion) were observed in a median of one joint (range 0–5) in each dog. Medial patellar luxation was diagnosed in 33.0% (8/24) of the evaluated dogs. At radiographic examination, elbow dysplasia was diagnosed in 48.2% (27/56) of elbow joints and severe hip dysplasia in 55.4% (31/56) of hip joints. The grade of elbow dysplasia was negatively associated with the ratio of static weight bearing between the front and hind limbs (slope estimate − 1.46, 95% CI − 2.75 to − 0.16, P = 0.03) and in dynamic weight bearing the ratio of total pressure index between the front and hind limbs (slope estimate − 0.088, 95% CI − 0.164 to 0.025, P = 0.03). The severity of hip dysplasia or hip osteoarthritis was not associated with the amount of static or dynamic weight bearing, but all except one dog were diagnosed with Fédération Cynologique Internationale grade C, D or E hips (dysplastic). In the spine, 78.6% (22/28) of the dogs had at least one malformed vertebra.

**Conclusions:**

Orthopaedic diseases and abnormal radiographic findings were common in the English bulldogs studied. The static weight bearing of the dogs was heavily distributed to the front limbs. With increasing severity of elbow dysplasia, the static and dynamic weight bearing shifted from dysplastic elbows to hind limbs.

## Background

The English bulldog is a popular dog breed worldwide. During the past decade awareness of the health problems of this brachycephalic breed has grown. Like most pedigree breeds, English bulldogs are selected for appearance by breed standard, and this has resulted in severe conformational abnormalities in these dogs [1]. Based on a review of pedigree dogs in the United Kingdom, the English bulldog ranks among the breeds with the highest prevalence of conformation-related disorders [1].

The most recognized health issues in English bulldogs have been related to foreshortening of the facial skeleton and brachycephalic obstructive airway syndrome (BOAS). Clinical signs include snoring, inspiratory stertor and stridor, exercise and heat intolerance, gastrointestinal problems and disturbed sleep patterns [2–4]. With increasing severity of BOAS grade, the exercise tolerance of these dogs decreases [4]. Many animals require surgical intervention for relief of their symptoms [2, 3]. However, brachycephalic syndrome is not the only problem suffered by this breed; musculoskeletal [1], dental [5], dermatological [6, 7] and ophthalmological [8] abnormalities have also emerged. Previous studies have reported an increased risk for hip dysplasia [9, 10], and the Orthopedic Foundation for Animals (OFA) has ranked the English bulldog as one of the breeds most prone to both hip and elbow dysplasia [11]. Patellar luxation [12, 13], congenital spinal malformations causing kyphosis and scoliosis, and spondylosis [14–17] have also been reported. In addition, the genetic background was recently revealed, with all English bulldogs found to carry a DVL2 gene mutation responsible for various skeletal malformations [18].

In orthopaedic research, it is currently common to evaluate dynamic musculoskeletal function of the dogs using kinetic or kinematic gait analysis [19–21]. Moreover, measurement of static weight bearing [22, 23] as well as measurement of range of motion in joints [24, 25] are often performed. Although several studies have reported various radiographic abnormalities in English bulldogs [1, 9, 10, 14–16], research evaluating the musculoskeletal function of the dogs and specifically the association of orthopaedic findings with dogs’ weight bearing and locomotion is scarce. Aristizabal Escobar et al. [12] assessed 30 English bulldogs with kinetic gait analysis and reported marked asymmetry in hind limb peak vertical force symmetry indices, although visual lameness was not detected. The authors suggested that the asymmetry was related to high prevalence of severe hip dysplasia in these dogs.

The aim of this study was to report the conformational, orthopaedic and radiographic findings in a cohort of English bulldogs in Finland and to evaluate how the conformation or various orthopaedic problems seen in these dogs affect their weight bearing and locomotion. Our hypothesis was that conformational, orthopaedic and radiographic findings would negatively influence dogs’ weight bearing and ability to move.

## Methods

### Recruitment and inclusion criteria of dogs

Privately owned, prospectively recruited pet dogs were enrolled in an English bulldog health study for respiratory, musculoskeletal, dermatological and exercise tolerance assessment. The study was conducted at the Veterinary Teaching Hospital, University of Helsinki, Finland between December 2014 and June 2015. The study plan was presented at the meeting of the national English bulldog breed club and announced on the websites of the breed club and the Veterinary Teaching Hospital. Inclusion criteria of the dogs were age of 2–5 years, registered in the Finnish Kennel Club (FKC) and no previous history of airway or orthopaedic surgery or any conditions increasing the risk of anaesthesia. Owners willing to take part in the study filled in the pre-study questionnaire on their perceptions of the dog’s physical activity and overall health. From the 54 answers received, 30 dogs with variable activity levels were chosen (one dog/owner) to participate in the study [7]. The study protocol was approved by the Committee of Experimental Animals, and all owners signed an informed consent.

### Evaluation of dogs

The dogs were evaluated at two visits. At the first visit, clinical, dermatological, orthopaedic and biomechanical evaluations and an exercise test were performed and blood samples taken. In addition to body weight, body condition score (BCS, range 1–5) was recorded. The dogs were expected to have elevated risk for upper airway disturbance and obstruction if their body temperature elevated above 39.3 °C. For safety, the evaluation was not done or was discontinued if the dog at the beginning or during the evaluation showed signs of dyspnoea or became hyperthermic. During the second visit, a maximum of 1 week later, dogs were anaesthetized, upper airways were visually evaluated and radiographic and computed tomography examinations were done. The results of dermatological and upper airway examinations, the exercise test and the computed tomography of the ears have been reported elsewhere [4, 7].

### Orthopaedic examination

An orthopaedic examination was performed by either of two experienced orthopaedic veterinary surgeons (SM, MM). The examination included lameness evaluation on a scale from 0 to 4 (no lameness, mild lameness or minor gait abnormality, moderate lameness or gait abnormality, severe weight bearing lameness, non-weight bearing lameness) [26] and palpation of the thoracic and pelvic limbs and spine as well as evaluation of conscious proprioception and withdrawal reflex. The spine was evaluated for pain and joints for pain, crepitation, swelling, decreased range of motion and instability (yes/no).

### Anatomical measurements

A physiotherapist specialized in animal physiotherapy (HH) measured the anatomical, static weight bearing and gait variables. While the dog was standing, distances from the ground to the highest point of the scapula (height at withers), olecranon process (height at olecranon) and greater trochanter of the femur (height at trochanter) were measured using a metric scale. Additionally, the width of the trunk (including soft tissues) at the widest region of the shoulder area (width of the trunk at scapula) and at the widest region of the hip area (width of the trunk at greater trochanter of the femur) was measured. To measure this, two vertically oriented bars placed to touch the widest region of the shoulder/hip area were held on both sides of the dog by the assistant, and the distance between the bars was measured with a metric scale. In addition to anatomic measurements in centimetres, front/hind ratio was calculated for the trunk width.

### Static weight bearing and temporospatial gait analysis

The distribution of static weight bearing between the limbs was quantitatively measured with a pressure-sensitive platform (Stance Analyzer, Petsafe, Knoxville, TN, USA). The dog was guided to stand on the measurement platform, each limb on its own quadrant of the plate. The owner was instructed to hold the dog from the front, keeping it in a straight square standing position and not to provide any manual support to the dog. The dog was held in place for 10–30 s, and a minimum of 10 consecutive measurements were obtained. To evaluate the effect of exercise and orthopaedic examination on the results, the measurements were taken three times: at the beginning of the visit, after the exercise test and gait analysis and, finally, after orthopaedic examination. The average ± SD values were calculated as percentages of total static weight bearing of all limbs and were reported for each limb as well as front and hindquarters of the animal. In addition, a ratio of the dog’s front and hindquarters (front/hind ratio) was calculated.

The temporospatial gait analysis was done using a pressure-sensitive portable walkway measurement system (GAITRite Electronic Walkway, Peekskill, NY, USA). The walkway had a 90*700 cm total area, and a scan rate of 240 Hz was used. The dog was guided by the owner over the walkway in both directions at a trotting speed. A trial was discarded if the dog was distracted, showed strong eye contact with the owner or was pulling on the leash. Three successful runs were collected, thus recording and interpreting at least 10 full gait cycles with the accompanied software. The evaluated variables were stride velocity (m/sec), stance time % of gait cycle (percentage of stance time of total gait cycle) and total pressure index percentage (sum of peak pressure values recorded from each activated sensor by a paw during mat contact/total sum of peak pressure values for all feet x 100). For these variables, also front/hind and left/right symmetry ratios were calculated. In addition, step length (distance between heel point of one foot and the heel point of the contralateral foot) was analysed.

### Radiographic examination and evaluation of the radiographs

For radiographic examination, the dogs were anaesthetized. The dogs were premedicated with butorphanole (Torbutor, Richter Pharma, Wels, Austria) intramuscularly 0.2 mg/kg, induced after preoxygenation with intravenous lidocaine (Lidocain, Orion, Espoo, Finland) 1 mg/kg and alfaxalone (Alfaxan, Dechra, West Sussex, Great Britain) to effect (approximately 2 mg/kg), and after intubation anaesthesia was maintained with sevoflurane (Sevorane, AbbVie, Espoo, Finland) and continuous infusion of lidocaine 2 mg/kg/h.

Radiographic evaluation of the elbow, hip and stifle joints was done by an experienced veterinary radiologist accredited by the FKC to grade hip and elbow dysplasia radiographs (AL). The spinal radiographs were evaluated by AL and KP, as a part of the licentiate thesis of the latter. In elbows, 45° flexed mediolateral views were used to evaluate the grade of elbow dysplasia on a scale from 0 to 3 (no, mild, moderate, severe) (FKC protocol based on International Elbow Working Group protocol) [27]. An extended ventrodorsal view of coxofemoral joints was used to evaluate the grade of hip dysplasia according to the Fédération Cynologique Internationale screening protocol, on a scale from A to E (grades A and B nondysplastic and C, D and E dysplastic with increasing severity) [28]. In addition, the amount of osteoarthritis (OA) of the coxofemoral joints was evaluated subjectively on a scale from 0 to 3 (no, mild, moderate, severe) and in the mediolateral and craniocaudal views of stifle joints on a scale from 0 to 3 (no, mild, moderate, severe) [29]. The laterolateral view of the cervical spine and the laterolateral and ventrodorsal views of the thoracic and lumbar spine were used to evaluate the shape and length of the vertebrae as well as the presence of spondylosis deformans. Malformed vertebrae were classified according to Gutierrez-Quintana et al. [15], and number and location of the malformed vertebrae were reported. Each vertebra was reported as negative or positive for spondylosis; no classification based on the type of finding was made. To evaluate spinal malformation-related kyphosis and scoliosis, Cobb angles were measured from laterolateral and ventrolateral views of the malformed vertebrae [14]. If more than one malformation was present, each was evaluated and the most significant change was selected for measurement of the Cobb angle. If no malformations were present, the measurement was taken from the 9th thoracic vertebra.

### Statistical analysis

Descriptive statistics were presented as mean ± SD values for the continuous variables. Frequency and percentage distributions or median (range) values were used for the categorical variables. Shapiro–Wilk test was applied to determine whether the data were normally distributed. Independent samples *t* test or Mann–Whitney test was used to detect differences between the sexes and between right and left limbs (when available).

The effect of radiographic findings on orthopaedic findings (yes/no) was analysed with mixed effects logistic regression, and the effect of radiographic findings on anatomic variables as well as on static weight bearing and temporospatial gait variables was analysed with linear mixed effect models using grade of the finding as a continuous variable. The effect of radiographic findings on static weight bearing ratio was analysed with analysis of variance model (ANOVA). Radiographic finding, limb (when available), gender and age were used as fixed factors in the models. The dog was included in the model as a random effect. In case of statistically significant effects, the effect was quantified with point estimates and their 95% confidence intervals (CIs) using odds ratios (ORs), slope estimates or differences of means depending on the type of model used. When the association of elbow and hip dysplasia findings with front/hind ratios was evaluated, combined single EDmax and HDmax values were used (grading result of the worse joint chosen). Statistical analyses were done using SAS System for Windows, version 9.3 (SAS Institute Inc., Cary, NC, USA). P-values < 0.05 were considered significant.

## Results

### Signalment

As two dogs could not participate due to acute illness, 28 English bulldogs were included in the study. The mean ± SD age and body weight, and median (range) BCS in 15 females were 3.5 ± 0.9 years, 22.0 ± 3.0 kg and BCS of 3 (2–4), and in 13 males 3.5 ± 0.9 years, 26.6 ± 3.3 kg and BCS of 3 (3–4), respectively. Based on the pre-study questionnaire, 24 owners (86%) reported that their dogs did not have any medical or other conditions that would affect the dog’s quality of life. In four dogs, furunculosis, eye and skin problems, OA or vomiting/regurgitation occasionally affected the daily quality of life, according to the owner. By the time of manuscript submission, according to the FKC database, 78.6% (n = 22) of the dogs had dog show results and 46.4% (n = 13) of the dogs had offspring, with a range of 1–7 litters.

### Orthopaedic examination

In four dogs, the orthopaedic examination was not done due to elevated body temperature and in three dogs, palpation in lateral recumbency could not be done due to increased dyspnoea or anxiety. In visual evaluation of lameness, unilateral grade 1 front limb and unilateral grade 1 hind limb lameness was seen in 20.8% (5/24) and 12.5% (3/24) of dogs, respectively. The pelvic limb conscious proprioception was decreased unilaterally in one and bilaterally in another dog, and pelvic limb withdrawal reflex was decreased bilaterally in one dog. One dog showed a pain response on palpation of the spine and tail, another on palpation of the lumbosacral junction and a third dog upon extension of the neck. Nine of 27 dogs (33%) had lesions of furunculosis in the interdigital skin, causing swelling and pain on palpation of the digits.

On palpation of the joints, three dogs showed at least one orthopaedic finding (i.e. pain response, crepitation, swelling, subjectively decreased range of motion) in the elbow. In coxofemoral joints, nine dogs showed at least one orthopaedic finding in hip palpation uni- or bilaterally. In stifle joints, at least one orthopaedic finding was detected uni- or bilaterally in 14 dogs. In addition, grade 1 medial patellar luxation was diagnosed unilaterally in one dog, grade 2 bilaterally in one dog and unilaterally in five dogs, and grade 3 bilaterally in one dog. In one dog, the tibial compression test was positive, indicating cranial cruciate ligament disease. When palpation findings of the elbow, stifle and coxofemoral joints were summed, orthopaedic findings were detected in a median of one joint (range 0–5) in each dog.

### Radiographic examination

Radiographs of all dogs were evaluated, and abnormal findings were common. At least grade 1 elbow dysplasia was diagnosed in 48.2% (27/56) of elbow joints, and in 10 of 17 affected dogs, the elbow joints were dysplastic bilaterally. The most common (55.4%) hip dysplasia grade was E (severe) and joints were dysplastic bilaterally in all dogs except the one dog with grade B hips bilaterally. Osteoarthritic changes in hip joints were diagnosed unilaterally in 3 dogs and bilaterally in 12 dogs. In stifles, 33.9% (19/56) of the joints had at least mild osteoarthritic changes, in 7 dogs bilaterally and 5 unilaterally. The results of elbow, hip and stifle radiographs are reported in Table 1. The dogs with an orthopaedic palpation finding in the stifle joint had an odds ratio of 1.43 (95% CI 1.11 to 1.85, P = 0.007) to have a finding also in radiographic examination of the stifle joint. No association was seen between orthopaedic and radiographic findings in elbow or hip joints.

**Table 1:** Radiological evaluation of elbow, hip and stifle joints in 28 English bulldogs

Elbow dysplasia					
Grade	0	1	2	3	
	29 (51.8%)	9 (16.1%)	15 (26.8%)	3 (5.4%)	
Hip dysplasia					
Grade	A	B	C	D	E
	–	2 (3.6%)	12 (21.4%)	11 (19.6%)	31 (55.4%)
Hip osteoarthritis					
Grade	0	1	2	3	
	29 (51.8%)	6 (10.7%)	21 (37.5%)	–	
Stifle osteoarthritis					
Grade	0	1	2	3	
	37 (66.1%)	14 (25.0%)	5 (8.9%)	–	

Number of joints (percentage of total number) in each grade class is reported. The grade of elbow dysplasia is reported on a scale from 0 to 3 (no, mild, moderate, severe) (FKC protocol based on International Elbow Working Group protocol), hip dysplasia on a scale from A to E (grades A and B nondysplastic and C, D and E dysplastic with increasing severity) (Fédération Cynologique Internationale screening protocol), and hip and stifle osteoarthritis on a scale from 0 to 3 (no, mild, moderate, severe)

**Fig. 1:**
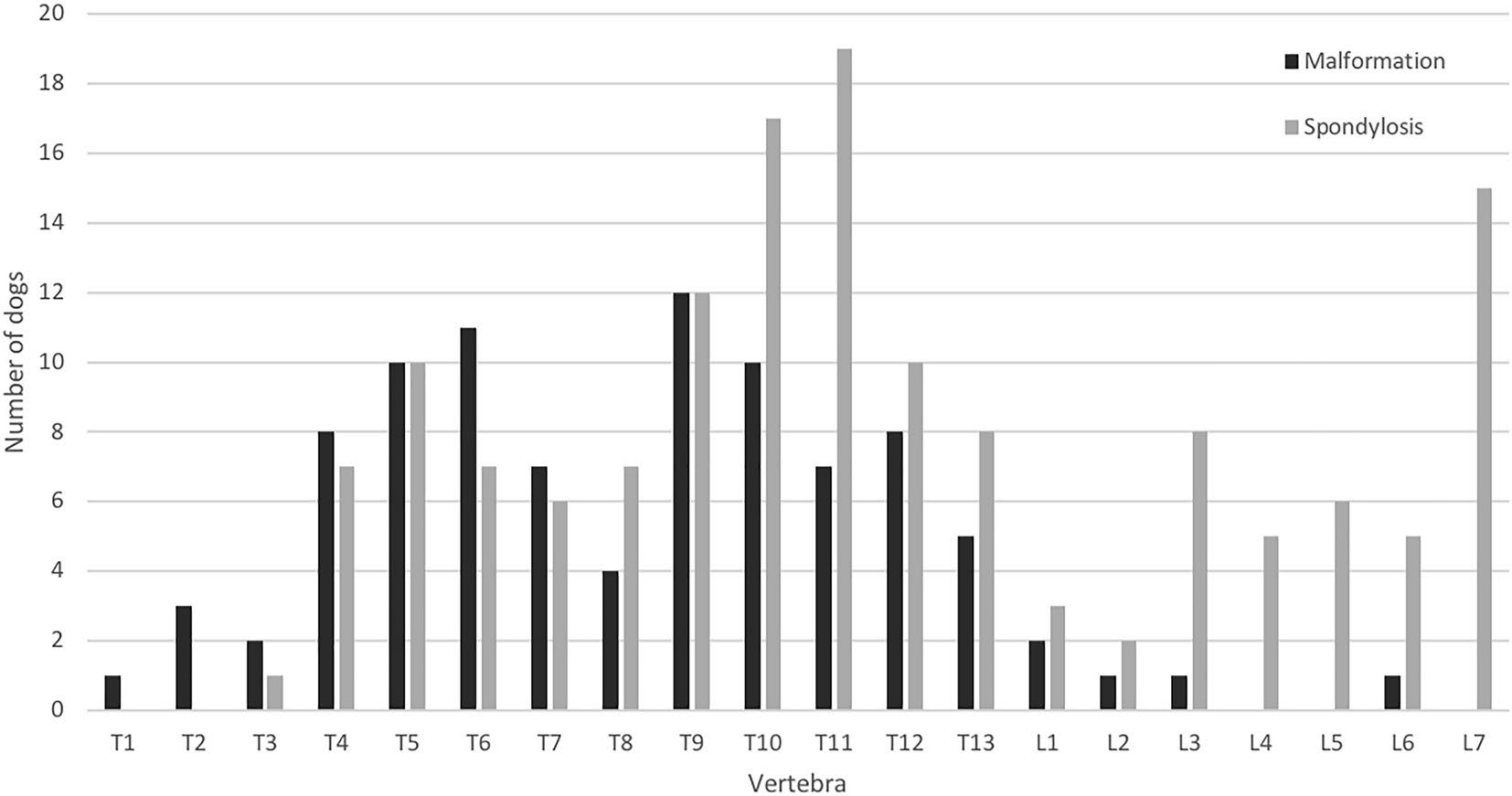
Number and distribution of malformed and spondylotic vertebrae in 28 English bulldogs

**Fig. 2:**
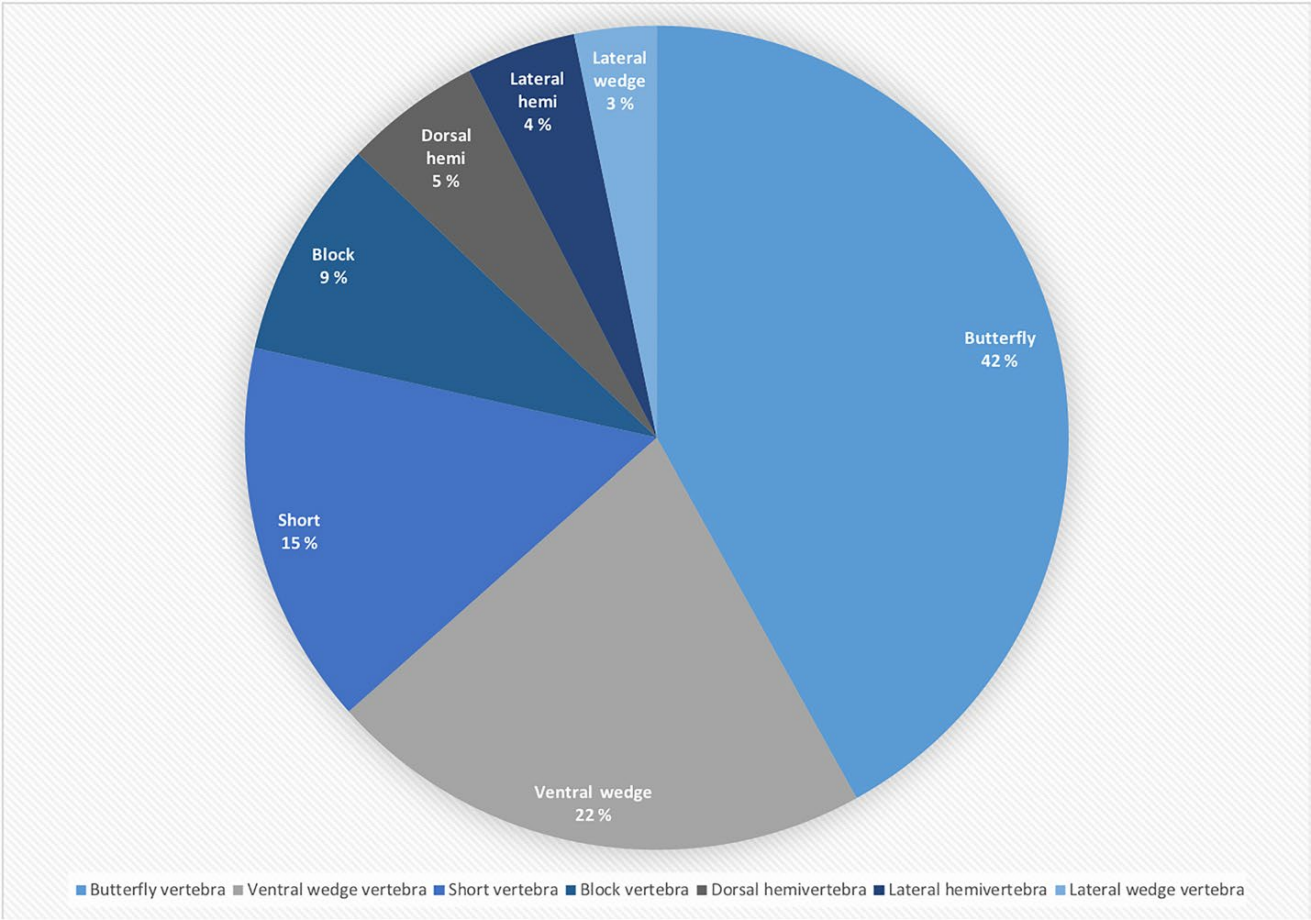
Percentage distribution of spinal malformation types in the thoracolumbar spine of 28 English bulldogs

A total of 756 vertebrae were evaluated for malformations, and 22 (78.6%) of all dogs had at least one malformed vertebra. Of cervical, thoracic and lumbar vertebrae, 2.0% (4/196), 24.5% (89/364) and 2.6% (5/196), respectively, were diagnosed as malformed. The median number (range) of malformations in C1–C7, T1–T13 and L1–L7 spine segments was 0 (0–2), 1.5 (0–6) and 0 (0–3), respectively. The distribution and types of malformations are reported in Figs. 1 and 2. The most frequently malformed vertebrae were T9 (n = 12), T6 (n = 11) and T5 and T10 (n = 10). Mean ± SD (range) Cobb angles were 9.9 ± 9.0° (0.4–32.5) for kyphosis and 6.4 ± 7.4° (0.1–34.6) for scoliosis. The largest mean Cobb angles were measured in vertebrae T4 (mean ± SD 15.1 ± 5.4°), T7 (14.9 ± 2.7°) and T9 (9.1 ± 3.9°) in lateral view and T10 (12.7 ± 14.8°), T4 (8.4 ± 6.4°) and T11 (8.4 ± 6.6°) in ventrodorsal view.

Spondylosis was diagnosed in 89.3% (n = 25) of dogs and in 20.2% (153/560) of vertebrae, resulting in a median number (range) of 4 (0–14) spondylotic vertebrae in each dog (Fig. 1). No significant difference emerged between females and males in the number of malformed vertebrae, number of spondylotic vertebrae or Cobb angles. However, the number of spondylotic vertebrae was positively associated with the age of the dog (slope estimate 1.82, 95% CI 0.35 to 3.29, P = 0.02). A positive association was seen between the number of malformed and spondylotic vertebrae (slope estimate 0.68, 95% CI 0.26 to 1.09, P = 0.002).

### Anatomical measurements

In one dog, anatomical measurements were not done due to its elevated body temperature. The results are reported in Table 2. The males were significantly taller and had significantly longer limbs than the females, but there was no difference between genders in width of the trunk or front/hind width ratio. Front/hind width ratio was negatively correlated with the grade of hip dysplasia (HDmax) (r = − 0.538, P = 0.005), and after removal of one outlier dog, dogs with grade E hip dysplasia (HDmax) had significantly smaller front/hind width ratios (mean 1.09, 95% CI 1.03–1.14) than dogs with grades B, C and D (1.21, 1.15–1.28), with an estimated difference of 0.130 (0.048–0.211, P = 0.003) between the groups. Also, a negative association was seen between the height at the trochanters and the grade of stifle OA (slope estimate − 0.66, 95% CI − 1.11 to − 0.21, P = 0.006, one outlier dog removed). Results from the statistical modelling presented here were obtained from the models excluding a single outlier dog. Statistical models including the outlier observation gave similar and statistically significant results, but the model residuals were not normally distributed.

**Table 2:** Anatomical measurements in English bulldogs (n = 27)

	Mean ± SD
Height at withers (cm)	39.5 ± 3.0
Height at olecranon (cm)	21.0 ± 1.4
Height at greater trochanter (cm)	35.8 ± 2.8
Width of trunk at scapula (cm)	25.3 ± 2.7
Width of trunk at greater trochanter (cm)	22.1 ± 3.0
Trunk width ratio front/hind	1.2 ± 0.1

### Static weight bearing and temporospatial gait analysis

In four dogs, static weight bearing was not measured at all due to elevated body temperature. The second (after exercise test and gait analysis) and third (after orthopaedic examination) measurements were available for 23 and 21 dogs, respectively. No significant differences were seen when results of each limb were compared between three measurement time points. The first measurement results of static weight bearing are reported in Table 3. While standing, English bulldogs bear a mean ± SD of 67.3 ± 3.1% of their body weight on the front limbs and 32.6 ± 3.0% on the hind limbs, with a front/hind ratio of 2.1 ± 0.3. The grade of elbow dysplasia (EDmax) was negatively associated with the ratio of static weight bearing between the front and hind limbs (front/hind ratio, slope estimate − 1.46, 95% CI − 2.75 to − 0.16, P = 0.03), indicating a shift of static weight bearing to the hind limbs with increasing severity of elbow dysplasia. Grades of hip dysplasia, hip or stifle OA or spinal abnormalities did not show any significant associations with the amount of static weight bearing.

**Table 3:** Static weight bearing measurements in English bulldogs (n = 24)

	Mean ± SD
SWB % F	67.3 ± 3.1
SWB % LF	32.9 ± 0.8
SWB % RF	34.5 ± 0.8
SWB % H	32.7 ± 3.1
SWB % LH	15.0 ± 0.6*
SWB % RH	17.6 ± 0.6*
SWB F/H ratio	2.1 ± 0.3

SWB % is calculated as the percentage of total static weight bearing values for all limbs. *SWB* static weight bearing, *LF* left front limb, *LH* left hind limb, *RF* right front limb, *RH* right hind limb, *F* front, *H* hind; *, significant (P = 0.003) difference in SWB between the hind limbs

For measurement of gait on a pressure-sensitive walkway, 22 dogs were able to complete the required trials for the analysis; only two trials were completed by one dog. For all evaluated dogs, the mean ± SD number of recorded gait cycles was 15.6 ± 2.5 and stride velocity 2.14 ± 0.27 m/sec. Stride velocity was significantly higher in males (2.27 ± 0.27 m/s) than in females (1.99 ± 0.19 m/s, P = 0.013). In trot, the mean ± SD sum of total pressure index percentage in front limbs was 62.1 ± 2.4% and in hind limbs 37.9 ± 2.4%, and the mean ± SD front/hind ratio of total pressure index was 1.65 ± 0.17. All results are reported in Table 4. There was a negative association between visually detected lameness and stride velocity (slope estimate − 0.30, 95% CI − 0.54 to − 0.07, P = 0.02).

**Table 4:** Results of temporospatial gait analysis in English bulldogs (n = 22)

	Mean ± SD
Stride velocity (m/sec)	2.14 ± 0.27
Stance time % of gait cycle LF	42.9 ± 2.9
Stance time % of gait cycle RF	43.0 ± 2.8
Stance time % of gait cycle LH	34.7 ± 4.0
Stance time % of gait cycle RH	34.5 ± 4.0
Stance time % of GC F/H ratio	1.25 ± 0.09
Stance time % of GC L/R symmetry	1.00 ± 0.03
Step length LF (m)	0.44 ± 0.07
Step length RF (m)	0.43 ± 0.05
Step length LH (m)	0.43 ± 0.07
Step length RH (m)	0.44 ± 0.05
Total pressure index % LF	30.7 ± 1.9
Total pressure index % RF	31.4 ± 2.0
Total pressure index % LH	19.0 ± 1.5
Total pressure index % RH	19.0 ± 1.6
Total pressure index F/H ratio	1.65 ± 0.17
Total pressure index L/R symmetry	0.99 ± 0.09

Total pressure index % is calculated as the percentage of total sum of peak pressure values for all feet. *LF* left front limb, *LH* left hind limb, *RF* right front limb, *RH* right hind limb, *F* front, *H* hind, *L* left, *R* right, *GC* gait cycle

When radiological findings were compared with the results of gait analysis, the grade of elbow dysplasia (EDmax) was negatively associated with the ratio of total pressure index between the front and hind limbs (slope estimate − 0.088, 95% CI − 0.164 to 0.025, P = 0.03), indicating a shift of dynamic weight bearing to the hind limbs with increasing severity of elbow dysplasia. The severity of hip dysplasia, hip or stifle OA or spinal abnormalities had no meaningful associations with measured gait analysis parameters.

## Discussion

The aim of this study was to report conformational, orthopaedic and radiographic findings in a cohort of English bulldogs and to evaluate how these findings affect the weight bearing and locomotion of the dogs.

### Hip dysplasia

As expected, severe hip dysplasia (grade E) was common; it was diagnosed in 55% of the hip joints evaluated. Only one dog was diagnosed with FCI grade B hips and all the rest had grade C, D or E. Our results are in accordance with the OFA screening statistics, where the English bulldog has been ranked the breed most commonly affected with hip dysplasia [11].

Hip dysplasia predisposes to joint inflammation and secondary OA, which can cause clinical disability and pain [30, 31]. The exact pathogenesis remains unclear, but hip joint laxity is assumed to be key for development of OA [32–34]. In addition, age, conformational characteristics and environmental influences, such as diet, have been reported to have a marked impact on the expression of OA in dogs susceptible to hip dysplasia [33, 35]. Although the majority of the evaluated dogs in our study had dysplastic hips, the amount of OA in hip joints was surprisingly low. Over 50% of the hips did not have any signs of OA. However, in a lifespan study of Labrador Retrievers, 43% of the dogs with subluxation of the hip joints had developed radiographic OA changes at only 5 years of age. By the end of life, all dogs had developed radiographic OA [36]. Although severely dysplastic, dogs in our study were only 2–5 years old during the evaluation, and thus, may develop OA signs later in life.

Progression and severity of OA changes have been shown to be delayed or minimized if the dog is kept lean [35, 37]. It has been suggested that excessive body weight and associated increased stress on joints transform the passive hip joint laxity to functional hip joint laxity, thus initiating OA [38]. The dogs in our study had a median body condition of 3/5, and thus, on average, were not obese. In addition, although the general body conformation of the breed is robust, broad and low in stature, most of the body weight is concentrated to the front of the dog, taking weight off the hind limbs. These factors also may have affected the absence of OA changes in our dogs.

Despite the high prevalence of severe hip dysplasia, only six dogs showed pain response on palpation of the hip joints, and four of these dogs also had signs of OA in the hip joints. Based on the English bulldog breed standard, the temperament of the breed is determined, intrepid, strong and energetic, but even-tempered. In the authors’ experience, English bulldogs as a breed are sometimes challenging to evaluate, and possibly some dogs did not show pain due to their stoic response to evaluation. Another explanation is that despite the severity of hip dysplasia the dogs were not in pain. The clinical course of hip dysplasia has been suggested to be often biphasic. In the juvenile form, clinical signs of lameness, bunny-hopping, reluctance to move and pain of the hip joints appear when the dog is between 5 and 12 months of age. These early acute signs are thought to be the result of extreme hip joint laxity. When the dog becomes older, the development of periarticular fibrosis is often associated with reduction or elimination of clinical signs. Later in life, clinical signs and pain may reappear, at this time most often related to development of degenerative joint disease [39, 40]. In our study, a large proportion of our 2- to 5-year-old dogs may have been in a clinically silent phase of hip dysplasia, therefore not showing significant pain or lameness during the evaluation.

We were unable to detect any correlation between the severity of hip dysplasia and the amount or distribution of weight bearing during standing or in trot. This was probably because we had only one dog with healthy hips and all other dogs had bilaterally dysplastic hips. Thus, we had insufficient variation to compare the musculoskeletal function in dogs with normal and dysplastic hips. However, during static weight bearing the weight of the dogs in our study was distributed mainly to the front limbs. Previously, it has been shown that dogs of average size and conformation bear approximately 60% of their weight on their front limbs and 40% on their hind limbs during both dynamic and static weight bearing [19, 21, 41]. In dogs of our study, 67% of the static body weight was on the front limbs and only 33% on the hind limbs. Although this breed is typically very robust and heavy in front, distribution of body weight to front limbs may also reflect discomfort or decreased function of hip joints due to poorly developed acetabuli as well as other orthopaedic problems in the spine or hind limbs. Interestingly, in trot the dogs distributed their weight more to the hind limbs, with 62% of body weight in front and 38% in hind limbs. The reason for this finding is unclear and may be related to the conformation of the dogs or a pain-related shift of body weight during increased loading of the limbs. Interestingly, in dogs with grade E hip dysplasia the anatomical front/hind width ratio of the trunk was smaller than in dogs with milder dysplasia grades. This is probably caused by a poorly developed acetabulum and inability of the femoral heads to stay reduced in the acetabular fossa in severely dysplastic joints, resulting in a wider distance between the major trochanters of the femur.

### Elbow dysplasia

Almost 50% of the elbows evaluated in our study were dysplastic, and in over 30% of elbows the dysplasia grades were two or three. In a recent study reporting kinetic gait analysis in English bulldogs, 20% of the dogs were diagnosed with elbow dysplasia and 10% also had degenerative joint disease in elbows [12]. Also, OFA screening statistics report that 36.6% of screened bulldogs were diagnosed with elbow dysplasia [11]. Similar to dysplastic hips, elbow dysplasia predisposes to secondary OA, pain and disability [42]. In our study, the visually detected front limb lameness and pain response to elbow joint palpation were reported only in 5 and 1 dogs, respectively. The low number of visually lame dogs is not surprising because visual evaluation has been found to be rather insensitive for detecting lameness [43, 44]. In addition, 59% of the dogs with elbow dysplasia were bilaterally affected, decreasing even more the possibilities for visual detection of lameness. However, the amount and distribution of both static and dynamic weight bearing was significantly associated with the elbow dysplasia grade. With increasing severity of elbow dysplasia, the amount of static weight bearing in front limbs decreased and in hind limbs increased, indicating a shift of body weight from dysplastic elbows to hind limbs during standing. In gait analysis, the front/hind total pressure index percentages were associated with the severity of elbow dysplasia, indicating a similar shift of weight bearing also in trot. The most likely reason for this is elbow dysplasia-related pain during weight bearing. According to our clinical experience, joint palpation in lateral recumbency does not necessarily cause a pain response in dogs with elbow dysplasia, but significant lameness or lift of weight off the limb may be seen when the limb is loaded.

### Patellar luxation

Median patellar luxation was diagnosed in 33% of stifle joints. In most dogs, grade 2 luxation was reported, but one dog had a grade 3 luxation in both stifle joints. In the study of Aristizabal Escobar et al. [12], 25% of the English bulldogs were diagnosed with bilateral patellar luxation, but the grade of the luxation was not reported. These findings are contrary to OFA screening statistics, where only 4.5% of screened dogs were diagnosed with patellar luxation [11]. However, in voluntary health programmes it is possible that the results are biased, as all abnormal results are probably not recorded in public databases.

### Spinal abnormalities

When radiographs of the spine were evaluated, 78.6% of the dogs had at least one malformed vertebra and 24.5% of the thoracic vertebrae were diagnosed as malformed. The most common type of malformation was the butterfly vertebra. The results are similar to a recent study where 75.6% of English bulldogs, 73.5% of Pugs and 93.5% of French bulldogs had one or more malformed thoracic vertebrae [16]. Spinal malformations have been commonly reported as an incidental finding, especially in “screw-tailed” brachycephalic breeds, and they may cause secondary kyphosis or scoliosis [14, 16]. The angular magnitude of the spinal deformity can be measured by using a Cobb angle [14]. The mean Cobb angles of the most deformed vertebrae in our dogs were 9.9° for kyphosis and 6.4° for scoliosis. Scoliosis has not been typically associated with neurological dysfunction, but kyphotic angle of 35° has previously been suggested as a cut-off value, above which the probability of associated neurological deficits is increased [14]. No dogs in our study approached this cut-off value, and none showed clinical signs of neurological dysfunction. Furthermore, no association was found between spinal findings and the amount or distribution of static or dynamic weight bearing. The development of spondylosis was strongly associated with the malformed vertebrae. As the spondylosis was located at the same locations as hemivertebrae, it could be speculated to be a supporting structure rather than caused by a degenerative process.

### Anatomical evaluation

In addition to radiographic evaluation and measurements of weight bearing, we also evaluated the conformation of English bulldogs. The published literature of body conformation in different dog breeds is scarce, usually limited only to simple measurements of height at the withers or simple distance measures regarding back or limb sections [45, 46], whereas front/hind width of trunk or width ratios are rarely measured. Knowing that English bulldogs are robust in front, we wanted to measure also the width of the trunk both in front and hind of the dogs, in addition to more commonly used height measures. Unfortunately, we were not able to compare our results to other breeds as there is no published literature on this subject. However, based on subjective evaluation of front/hind width ratios in our dogs, English bulldogs seem to be broad in their front, narrow in their hind or both. This also may partly explain the finding that these dogs bear more weight on their front limbs than other dog breeds. Whether the subjectively high front/hind width ratios are simply due to the anatomical conformation of English bulldogs or disease related changes to muscular or skeletal system, remains unclear and further studies are needed to evaluate this.

### Limitations

There are several limitations in our study. First, the number of dogs was low; only 28 dogs were enrolled. Dogs with variable activity levels were chosen to participate in the study, thus trying to include a comprehensive sample of the English bulldog population in Finland. The selection was done based on an owner-filled pre-questionnaire and it is possible that owners with several dogs enrolled their healthiest dog in the study, knowing that the intention is to study a breed that has received a lot of negative publicity. However, if the population was biased in this way, the results would then be better than the actual situation in the whole population. In addition, the results of this study only apply to Finnish English bulldogs, and thus, cannot be generalized to include the whole Bulldog breed. However, similar findings have been reported in other countries.

Second, not all evaluations were performed on all dogs due to the decreased tolerance to exercise or handling. Approximately 25% of the dogs did not tolerate the orthopaedic evaluation in lateral recumbency due to dyspnoea or anxiety, or the kinetic gait evaluation due to abnormally elevated body temperature. Although not directly related to the musculoskeletal health of the dogs, these findings support the perception that a significant proportion of English bulldogs are unable to carry out the behaviour typical for the canine species such as normal locomotion and function in everyday life.

As a limitation in kinetic gait evaluation, the dogs were allowed to move at their natural speed and no velocity limitations were applied. The velocity has been shown to affect the gait analysis parameters, and narrow velocity limits are recommended to minimize the bias in data values. In our study, we chose not to limit the velocity in order to increase the probability of gathering enough trials for the analysis. Using the velocity limits would have required a larger total amount of trials and probably resulted in more dogs dropping out from the evaluation due to exercise intolerance and elevated body temperature. In addition, the dogs were evaluated only once, no serial measurements with differing velocities were compared, and most of the gathered data was analysed as percentages or ratios, thus minimizing the influence of velocity on gait parameters. An interesting finding was that the dogs diagnosed visually as lame trotted at a slower velocity than the dogs not showing any visually detected lameness.

## Conclusions

Orthopaedic and radiographic findings were common in English bulldogs of our study, with a considerable number of dogs having hip and elbow dysplasia, patellar luxation and spinal abnormalities. Unlike in other previously reported breeds, the static weight bearing of the dogs was heavily distributed to the front limbs. We were able to confirm our hypothesis that orthopaedic diseases, especially elbow dysplasia, affect the weight bearing and locomotion of English bulldogs.

For the future of the English bulldog breed in Finland, it seems unlikely that any changes in breeding could produce healthier individuals when taking into account that the prevalence of orthopaedic diseases is high and in some conditions like hip dysplasia, no healthy individuals exist. In addition, orthopaedic problems are not the only condition that plagues this breed. At this point, the chances for selective breeding are lost and probably the only option towards healthier dogs would be crossbreeding.

## Data Availability

The datasets used and/or analysed during the study are available from the corresponding author on reasonable request.
